# Atypical polypoid adenomyoma follow-up and management: Systematic review of case reports and series and meta-analysis

**DOI:** 10.1097/MD.0000000000020491

**Published:** 2020-06-26

**Authors:** Anna Biasioli, Ambrogio P. Londero, Maria Orsaria, Federica Scrimin, Francesco Paolo Mangino, Serena Bertozzi, Laura Mariuzzi, Angelo Cagnacci

**Affiliations:** a Clinic of Obstetrics and Gynecology, University Hospital of Udine; b Ennergi Research; c Institute of Pathology, University Hospital of Udine; d Institute for Maternal and Child Health, IRCCS “Burlo Garofolo”, Trieste (TS); e Department of Medical Area (DAME), University of Udine, Udine (UD); f Clinic of Obstetrics and Gynecology, DINOGMI, IRCCS San Martino Hospital, University of Genova, Genova (GE), Italy.

**Keywords:** atypical polypoid adenomyoma, endometrial cancer, eneometrial hyperplasia, fertility sparing treatment, operative hysteroscopy

## Abstract

**Background:**

Atypical polypoid adenomyoma (APA) is a rare uterine tumor typically found in fertile age and associated with infertility. Among young nullipara women, conservative treatment is proposed despite the high recurrence rate and the association with endometrial cancer.

Our aim was to assess the risk of recurrence with different conservative treatments in fertile ages and the prevalence of malignant or pre-malignant associated lesions to better address an adequate patient counselling when treatment modalities are discussed.

**Methods:**

This study is a systematic review and meta-analysis of case reports and case series about APA management and follow-up. A literature search was carried from Medline and Scopus for studies published from January 1, 1980 to December 31, 2018.

**Results:**

We included 46 observational studies and 296 cases in fertile women. The prevalence of APA relapse was 44% (CI.95 33–57%) and was lower in cases treated with operative hysteroscopy (22%; CI.95 11–39%) than in cases treated with blind curettage and polypectomy (38%; CI.95 15–67%). The prevalence of the concomitant or during the follow-up diagnosis of endometrial carcinoma was 16% (CI.95 9–29%). The risk of cancer development during follow-up was significantly less in cases treated with histeroscopy (10.56% new cumulative diagnosis at 5 years follow up; CI.95 0–23.7%) than blind curettage and polypectomy (35.5% new cumulative diagnosis at 5 years; CI.95 11.65–52.92%; *P* < .05). Medical treatment with medroxyprogesterone acetate after surgery does not reduce APA recurrence. Pregnancy was observed in 79% cases in which the desire was expressed.

**Conclusion:**

This review suggests that conservative treatment performed by operative hysteroscopy is the optimal choice because it lowers the risk of recurrence, improves the accuracy of concomitant carcinoma or hyperplasia diagnosis, and leaves the possibility of future pregnancies.

## Abstract 2

Supplemental Digital Content is available in the text

**Highlights - Whats this paper adds**

- Conservative treatment of atypical polypoid adenomyoma performed by operative histeroscopy is the optimal choice because it lowers the risk of recurrences.
- Operative histeroscopy improve the accuracy of concomitant carcinoma or hyperplasia diagnosis.
- The medical treatment with medroxy progesterone acetate, following conservative surgery, does not appear to be beneficial in reducing atypical polypoid adenomyoma recurrences.
- The persistence/recurrence of atypical polypoid adenomyoma at 10 years follow up was 59.54% (CI.95: 32.88–75.61%).
- Concomitant diagnosis alone of endometrial carcinoma was 11% (CI.95 7–17%) and endometrial carcinoma during follow up was 14% (CI.95 7–26%), and the time-to-event cumulative events were 59.91% (CI.95: 29.54–77.19%) at 14 years of follow-up.

## 1 Introduction

Atypical polypoid adenomyoma (APA) is a rare uterine tumor composed of atypical endometrial glands surrounded by smooth muscle tissue bundles. It is intended as benign epithelial and mesenchymal neoplasm, but it can lead to diagnostic difficulties given the degree of atypia that distinguishes it. The term APA was introduced by Mazur in 1981 which described 5 polypoid lesions in premenopausal women characterized by atypical glands with squamous metaplasia and a smooth cellular muscle stroma, previously classified differently from other authors.^[1]^ At an initial assessment, the picture may appear as adenocarcinoma or mixed Mullerian tumor, and the diagnosis may be complex in some cases.^[1]^

APA is a rare disease, there are <500 cases reported in the literature and therefore it is difficult to evaluate its incidence. Typically it is found in fertile age, but some cases are described also in post-menopausal period.^[2,3]^ In most cases patients present with abnormal uterine bleeding, menometrorrhagia, or with anemia.^[4]^ The diagnosis of APA is essentially histological and it is often macroscopically and clinically indistinguishable from endometrial polyp, submucous myoma, or adenofibroma.

In most cases it is found in young women with possible associated infertility. Therefore, conservative treatment has been considered as a valid choice, but recurrences are common as well as the coexistence or development of atypical endometrial hyperplasia or endometrial cancer. The origin for the majority of APA cases (80%) is recognized at the cervical/isthmus level but it can originate in all uterine portions.^[5]^

Because APA is rare pathology and affected cases are often presented as single case or small series, aggregation of these cases by systematic review and meta-analysis may help to understand its appropriate clinical management.^[6–8]^

The underlying question guiding this review was if the conservative management of APA is a safe procedure in women of childbearing age with pregnancy desires. Accordingly, we performed a meta-analysis to assess the concomitancy (or new diagnosis during follow up) of pre-malignant or malignant lesion, and recurrences consequent to different APA clinical managements (Supplemental Table 1).

## 2 Materials and methods

This study is a systematic review and meta-analysis of case reports and case series about APA management and follow-up. In addition, the PRISMA guidelines (Preferred Reporting Items for Systematic Reviews and Meta-Analyzes) were followed for the drafting of this meta-analysis.^[9]^

### 2.1 Search strategy for review

A literature search was independently carried out by 2 authors (AB and APL). All information was gathered from Medline and Scopus for studies published from January 1, 1980 to December 31, 2018 (by online search engines). The following query was used to search the Medline/PubMed database: “atypical[All Fields] AND polypoid[All Fields] AND (”adenomyoma“[MeSH Terms] OR ”adenomyoma“[All Fields])”; while Scopus was queried using the following strategy: “TITLE-ABS-KEY (atypical AND polypoid AND adenomyoma).” Meta-information, titles, and abstracts resulted from these queries were examined. All articles that referred to APA management were selected and full texts were searched and analyzed. Eventually, bibliographies and citations from Scopus items, previous review publications, and full articles were used to identify other potentially relevant articles.

### 2.2 Eligibility criteria, study selection, and data collection process

All studies that evaluated management in patients affected by APA were considered. Only studies about women of childbearing age presenting information on management and follow-up concerning relapses, diagnosis of endometrial hyperplasia, diagnosis of endometrial cancer, and pregnancy were included. In addition, only studies performed on human subjects where a full text was available for data retrieval and written in English were included (we did not contact any authors of the studies for further information). All articles not meeting the inclusion criteria as stated above were discarded. Moreover, editorials, letters to the editor without original data, and reviews were excluded. In addition, conference abstracts were excluded due to the lack of details regarding data to assess the methodological quality.

The resulting items after database queries were checked for duplicates and all titles and abstracts were screened to select all the articles that met the inclusion criteria. Afterwards, the full articles of the selected items were explored and reference lists, citations, and previous review publications were searched to identify other additional pertinent articles. Hereafter, 2 reviewers (AB and APL) independently selected the studies and extracted data from the full-text articles. Among other data (patients characteristics, eventual medical treatment, adenomyosis, follow up time, eventual APA recurrence and time at recurrence, diagnosis of endometrial cancer or endometrial hyperplasia with atypia during the follow up and at the time of diagnosis, pregnancy desire, and successful pregnancy), patient treatment time frame, geographic locations, and type of treatment were retrieved from full text articles in order to avoid any possible overlap of cases. When two or more studies used the same set of patients or presented possible data overlap, only the one that was more recent and of better quality or with more detailed data was included (if 2 articles presented different aspects of the same case both articles were included and merged for the analysis). Any discrepancy was addressed by a joint re-evaluation of the original article. For the purpose of this study we stratified the conservative surgical treatment in 2 categories: the classical inpatient blind uterine dilatation, curettage, and polypectomy (DCP) including also cervical polipectomy with forceps, that has been the technique routinely used for many years to perform uterine polypectomy; and operative histeroscopy (OH) (resection of the polyp and biopsies under vision).

### 2.3 Assessment of methodological quality for included studies

The quality of the literature has been evaluated through the use of the CARE check list, in particular the presence of the following items was assessed: patient information, clinical findings, the presence of timeline information, diagnostic assessment, therapeutic intervention, follow-up, and outcomes.^[10]^ We considered the presence (+) of the information concerning a specific item to be a sign of study with a low risk of bias, while the absence (–) of an item was considered a sign of a study with a high risk of bias. In some cases the item was classified as unclear (?) due to the absence of sufficient information.

### 2.4 Data analysis

The analysis was performed using R (version 3.5.3 - R: A language and environment for statistical computing. R Foundation for Statistical Computing, Vienna, Austria), a *P* < .05 was considered as significant. We calculated a summary statistic of the prevalence of the outcomes taken into consideration. The *I*^2^ index and the Cochran Q are used to evaluate heterogeneity between studies. An index value of *I*^2^ > 50% and a value of *P* of Cochran *Q* <0.10 were considered statistically significant signs of heterogeneity. Where appropriate, the fixed or random effects models were applied to calculate the synthesis estimate. In addition case series with <5 cases and case reports were aggregated for the analysis in forest plots. We also performed a Kaplan–Meier analysis of cumulative events as previously described.^[7]^ The risk of bias assessment was not possible for this study. This case report and case series meta-analysis is exempt from ethical approval as the analysis involves only already published and anonymized data.

## 3 Results

### 3.1 Search Results

**Figure 1:**
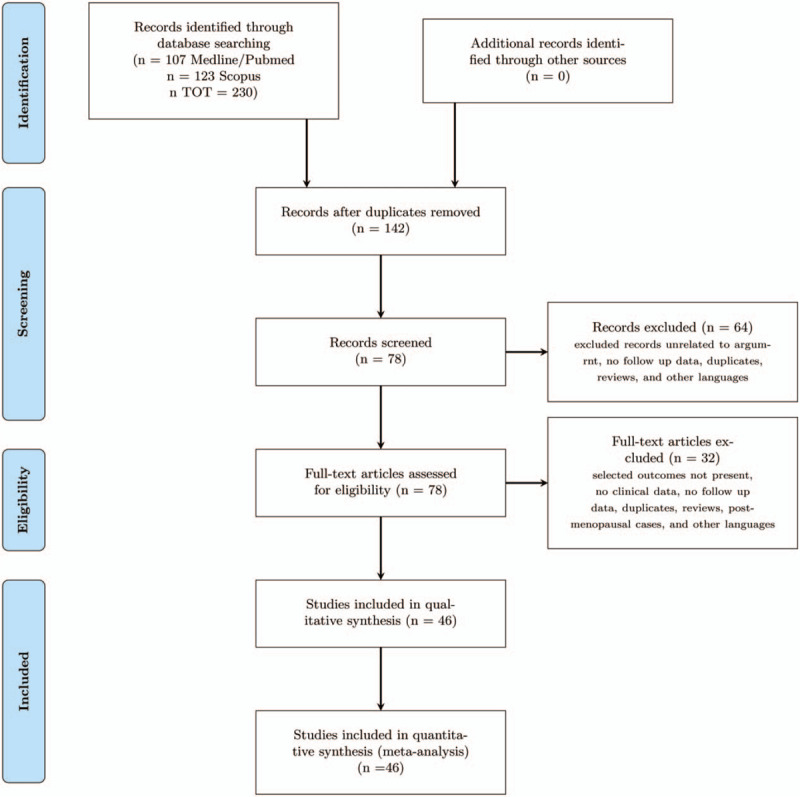
Flow chart of the study.

The literature search flowchart is shown in Fig. 1. Overall 142 studies were found (Fig. 1) and 64 items were found to be not eligible after reviewing the titles and the abstracts. Hence the potentially eligible articles for this review were in total 78. However, of these potentially eligible items for 2 articles it was not possible to obtain the full text and for other 30 of these articles inclusion criteria were not matched. In the Supplemental List 1, we show the included and excluded studies. We finally selected 46 eligible articles (Fig. 1, Supplemental Table 2, and Supplemental List 1). All these 46 articles included in this review were case reports or case series (only 10 articles comprise >5 cases).

### 3.2 Characteristics of the included studies

In our systematic review and meta-analysis, we included 46 observational studies that evaluated APA management in fertile women. The main characteristics of these studies are reported in Supplemental Table 2. Of these 46 studies, 41 presented data for individual patients for a total of 129 cases (therapeutic management and follow up of 1 case was presented in 2 different items a case report and a letter to the Editor).^[11,12]^ From another 6 studies with 202 cases, the data were aggregated. In one study of 35 cases only some information was extracted for the individual patients and this study was considered in both the previous groups.^[13]^ Therefore in total, 296 cases of APA were considered in this systematic review of the literature.

Hereafter, we report the epidemiological and clinical features extrapolated from the 127 cases with data for single patient. Considering all 129 cases, the median age of fertile patients with APA was 35 years (IQR 29–40), the BMI present for 45 patients has a median value of 21 kg/m^2^ (IQR 19–24). Based on the reported data, the patients were mostly nulliparous (93.69% of cases [104/111]) and with a history of infertility in 61.76% of cases (21/34). Symptoms such as abnormal uterine bleeding, were present in the majority of cases 76.12% (51/67).

All patients underwent primary surgical treatment: in most cases conservative. In fact, 19/94 (20.2%) underwent hysterectomy as first treatment after diagnosis (these were mainly old patients and in any case in older studies). In 79.9% of cases a conservative treatment has been proposed: blind techniques such as polypectomy or cavity revision in 42.4% of cases and operative hysteroscopy in 37% (34/92) but with an increase in prevalence in more recent years. In addition, recently also laparoscopic and lapatotomic conservative approach has been proposed.^[14,15]^ If a follow-up treatment was described for recurrence, persistence or appearance of malignant lesions, 21 further hysterectomies were performed on 27 reported cases. Post-surgery medical treatment was performed in 35/129 patients (27.1%), in 33 cases (94.3%) was used medroxy progesterone acetate (MPA) in varying patterns and doses, and in 2 cases (5.7%) levonorgestrel medicated IUDs was used (in 1 case as adjuvant therapy after first treatment and in 1 case after the treatment of the second recurrence, none of these 2 cases had a recurrence). In 2 cases was described >1 recurrence but for the purpose of this study we considered only the first recurrence.

There were 5 studies only with aggregated data.^[16–20]^ In these case series the average age ranged between 38 and 56 years, with a prevalence of nulliparous from 50% to 76% and a prevalence of infertility from 15% to 35%. In these 5 studies the treatment was prevalently conservative with a median of 76% of cases treated in a conservative way, a minimum of 46%, and a maximum of 86.4%.

### 3.3 Methodological quality assessment of the included studies

**Figure 2:**
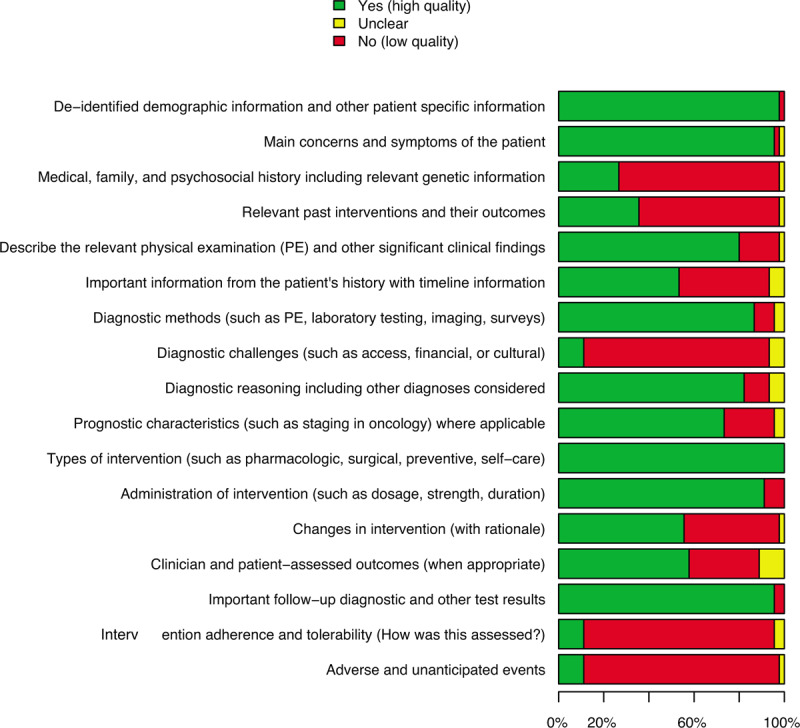
This chart on methodological quality shows the summary, considering all included studies in this meta-analysis, of each specific methodological quality item.

Figure 2 and Supplemental Fig. 1A and 1B highlight the methodological quality of the articles included in this systematic review and meta-analysis of the literature. Figure 2 presents a summary of the evaluation of the items considered (the detail of the evaluation of each item for the various studies is shown in Supplemental Fig. 1A and 1B). Most items exceed 50% of positivity (the information is present and therefore we suppose a low risk of bias). There was no information on conflict of interest or no significant conflict of interest and any declared funding or any funding by commercial agencies in the examined articles. The publication bias could not be examined because it is not applicable in this case.

## 4 Main results

### 4.1 Analysis of APA recurrences

**Figure 3:**
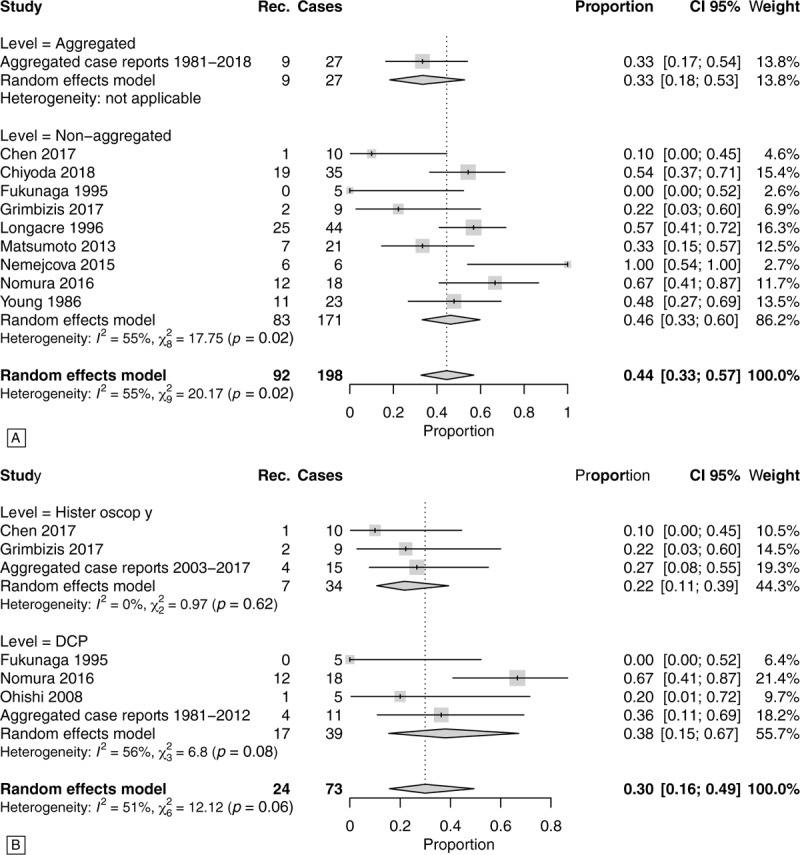
Summary forest plots of APA recurrences without considering the specific time-to-event follow-up. Panel A: Forest-plot of APA relapse prevalence. Case series with <5 cases and individual case reports were aggregated. Panel B: In this forest-plot data were stratified by type of conservative treatment DCP versus operative histeroscopy (for this analysis we did not stratified between aggregated and non-aggregated data). Case series with <5 cases and individual case reports were aggregated. APA = atypical polypoid adenomyoma, DCP = dilatation, curettage and polypectomy.

The prevalence of APA relapses during follow-up was 44% (CI.95 33–57%) on 198 cases analyzed (hysterectomies were excluded because this outcome could not be evaluated) (Fig. 3A). In Fig. 3A, that shows the forest plot of the APA recurrence prevalence, there is an important heterogeneity in the data. To justify this heterogeneity we considered the possibility of different follow-up lengths and of different treatment approaches (blind DCP vs operative hysteroscopy or use of “adjuvant” medical therapy).

Therefore, an analysis was performed on the prevalence of relapses according to the type of surgical treatment: DCP versus resection under hysteroscopic vision (Fig. 3B and Supplemental Fig. 2. The prevalence of relapses was lower if the conservative approach was that of operative hysteroscopy rather than DCP. In an analysis by stratifying between operative hysteroscopy versus DCP we obtained a prevalence of relapses of 22% (CI.95 11–39%) in cases treated with operative hysteroscopy versus 38% (CI.95 15–67%) in cases treated with RCU/polypectomy (Fig. 3B).

**Figure 4:**
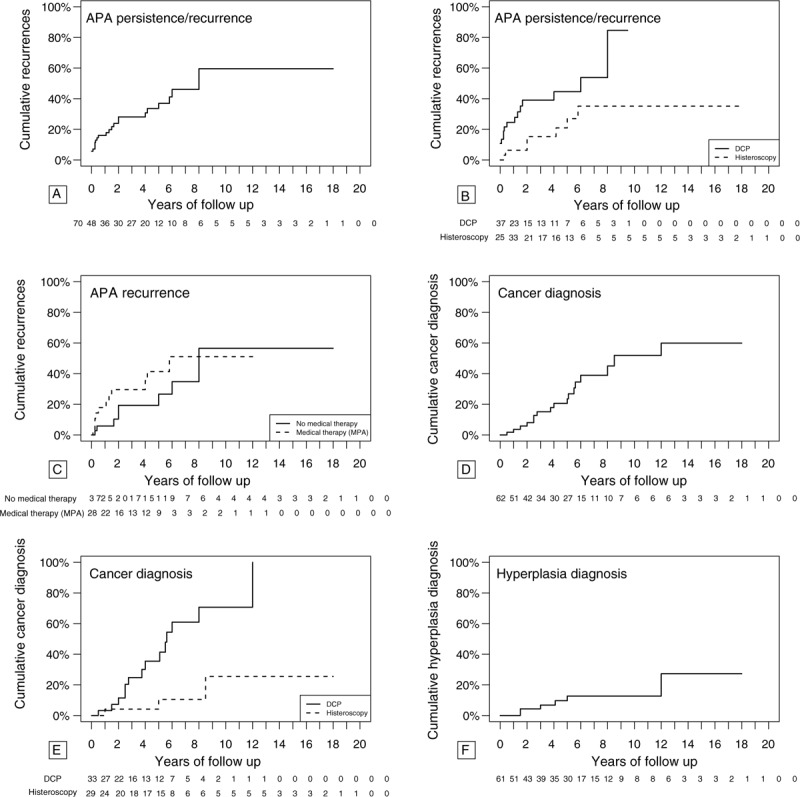
Kaplan–Meier analysis based on the follow-up time in cases treated conservatively and in which the time-to-event data in the follow-up was available. Panel A: Analysis considering the cumulative APA relapses. Panel B: Analysis considering cumulative APA recurrences and type of conservative treatment (DCP vs operative histeroscopy Log-rank test *P* < .05). Panel C: Analysis considering cumulative APA relapses: in this plot cases treated with medical therapy after surgery were compared with cases not treated with medical therapy after surgery (Log-rank test *P* < .05). Panel D: Analysis considering the cumulative endometrial cancer diagnoses. Panel E: Analysis considering the cumulative endometrial cancer diagnoses: data were divided in 2 groups according to the type of conservative treatment (DCP vs operative histeroscopy Log-rank test *P* < .05). Panel F: Analysis considering the diagnosis of cumulative endometrial hyperplasia. APA = atypical polypoid adenomyoma, DCP = dilatation, curettage and polypectomy.

The analysis of the cumulative relapses on the 70 cases in which it was possible to extrapolate the data on time-to-event follow-up, we found that the risk increases with the increase of follow-up length and at 10 years follow up was 59.54% (CI.95: 32.88–75.61%) as high as in the studies with greater follow up such as Longacre et al^[19]^ or Chiyoda et al^[13]^ (9–10 years) (Fig. 4A). It was also statistically significant, during the follow-up, a greater tendency to persist or to relapse in cases treated with DCP compared with operative hysteroscopic treatment (*P* < .05) (Fig. 4B).

Post-surgical progestogen medical treatment was used in a small percentage of cases (26.8% of the total) and only in 5 studies.^[4,15,21–23]^ The most common treatment was MPA. Supplemental Fig. 3 shows no protective effect of MPA against recurrence in cases where medical therapy was given after surgical treatment (26% vs 42%). In Fig. 4C relapses are not significantly different between the MPA treated and non-treated cases (persistent cases were excluded from this analysis). In only 2 cases the medicated IUD with levonergestrel was used, and in one of these cases the IUD was used after the appearance of the second recurrence.^[15,23]^

### 4.2 APA association with endometrial neoplasia and atypical hyperplasia of the endometrium

**Figure 5:**
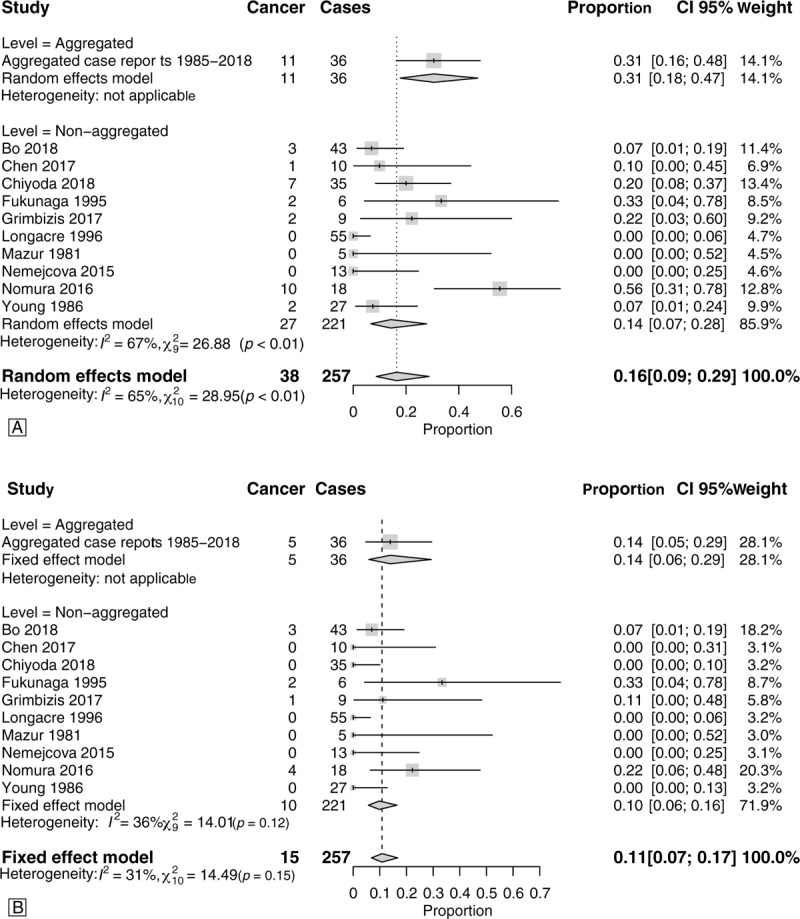
Summary forest plots of endometrial cancer diagnosis. Panel A: Forest-plot of the prevalence of concomitant or during the follow-up diagnosis of associated endometrial neoplasia. Case series with <5 cases and individual case reports were aggregated. Panel B: Forest-plot of the prevalence of concomitant diagnosis of associated endometrial neoplasia. The time-to-event follow-up was not considered in this analysis. Case series with <5 cases and individual case reports were aggregated.

The prevalence of the concomitant diagnosis or the diagnosis during the follow-up of endometrial carcinoma was 16% (CI.95 9–29%) (Fig. 5A). The concomitant diagnosis alone of endometrial carcinoma was 11% (CI.95 7–17%) (Fig. 5B). The diagnosis of endometrial carcinoma during follow up was 14% (CI.95 7–26%) (Supplemental Fig. 5). There was a greater uniformity in the prevalence of diagnosis of concomitant endometrial cancer among cases undergoing operative hysteroscopy compared with blind treatment (Supplemental Fig. 4).

Evaluating the time course, the risk of diagnosis of endometrial neoplasia was 59.91% (CI.95: 29.54–77.19%) at 14 years of follow-up (Fig. 4D). The risk of cancer development during follow-up according to the cumulative analysis is statistically less prevalent in cases treated primarily with operative hysteroscopic surgery (10.56% new cumulative diagnosis at 5 years follow up; CI.95 0–23.7%) than in cases treated with DCP (35.5% new cumulative diagnosis at 5 years; CI.95 11.65–52.92%; *P* < .05) (Fig. 4E).

Figure 4F, and Supplemental Figs. 6, 7 and 8, show the association with endometrial hyperplasia at the time of diagnosis or during follow-up. From the literature analysis, the association with endometrial hyperplasia is 15%, the diagnosis is concomitant in 10% of cases and occurs in follow-up in 11% of cases.

Concerning the cumulative analysis, the risk of diagnosis of endometrial hyperplasia is 27.27% (CI.95: 0–50.15%) at 14 years of follow-up (Fig. 4F). No significant risk differences were found considering different conservative surgical management.

### 4.3 Pregnancy and adenomyiosis

Data on pregnancy outcome are reported in a low percentage of studies. This depends above all on the fact that older studies probably do not take into account the preservation of fertility, while there is an absolute tendency in recent years. However, pregnancy success is reported in 15/19 (79%) cases in which the desire was expressed.

Finally, the association with adenomyosis is 18% (CI.95 8–37%) (Supplemental Fig. 9).

## 5 Discussion

APA is a rare condition and there were <500 cases among the eligible studies for this systematic review. Therefore, it is difficult to hypothesize in the future the feasibility of prospective or randomized studies. There are case reports or case series and only 10 of these include >5 cases.^[1,4,13,16–20,24–26]^ In the literature it is stated a clear role for aggregated case reports to increase the understanding of the trajectories of care in rare conditions. Therefore, case reports meta-analysis are found to be an important instrument in rare conditions where clinical studies are not a practicable option.^[6–8]^ Recently a meta-analyses from case reports was claimed to have overlapping results with a meta-analyses from clinical studies.^[6–8]^

The main result in our study was an APA relapse prevalence of 44% (CI.95 33–57%) and a significant lower prevalence in cases treated with operative hysteroscopy (22%; CI.95 11–39%) than in cases treated with DCP (38%; CI.95 15–67%). Also the risk of cancer development during follow-up was significantly less in cases treated with hysteroscopy (10.56% new cumulative diagnosis at 5 years follow up; CI.95 0–23.7%) than DCP (35.5% new cumulative diagnosis at 5 years; CI.95 11.65–52.92%; *P* < .05). Pregnancy was observed in 79% cases in which the desire was expressed.

The association of APA with adenomyosis was 18%, however only in a minority of cases this information was reported in the studies, especially in older reports it was probably under-reported due to limited diagnostic accuracy of non-invasive methods at that time. The lesion mainly arises in fertile age with a median age of 35 years, in infertile or otherwise nulliparous women. Infertility is found in 61.76% of cases where information was reported and nulliparous represent 93.69% of these cases.

The high prevalence of nulliparous women with subfertility underlines the need to establish the safety of a conservative treatment for a lesion considered benign but for which the data in the literature show a risk of recurrence, and of association with endometrial hyperplasia and cancer. Heatley^[27]^ in a 2006 review points to a risk of 30% relapse and a 9% risk of association with cancer and hypeplasia. However, these data are older than 10 years ago, they do not seem to emerge from a systematic review of the literature and above all do not take into account the most recent treatment experiences in hysteroscopic surgery. This systematic review of the literature and the meta-analysis performed may be useful in the management of these rare lesions in a particular patient setting as described above.

In 79.79% of cases a conservative treatment with preservation of fertility has been proposed, while histerectomy is restricted to cases in post-menopausal period (not included here) or as first line treatment in older studies or in pre-menopausal age. The risk of recurrence of APA in this meta-analysis is 44%, with a conspicuous heterogeneity between studies and peaks, especially in the larger case series that evidently include longer recruitment and follow-up periods. And in this meta-analysis the time-to-event analysis showed a 10 years cumulative recurrence of 59.54%.

The hypothesis underlying this work, however, is linked to the fact that different frequencies of relapse depend on the type of treatment performed. Already Matsumoto et al^[18]^ showed in his case series a risk of recurrence of 36% in patients treated with DCP and 10% in patients undergoing hysteroscopic removal. Our meta-analysis confirmed this difference by reporting a risk of persistence/recurrence of 38% versus 22% respectively in the case of DCP or hysteroscopic treatment (Fig. 3B). Confirmed also by the time-to-event analysis (Fig. 4B).

Medical treatment with progesterone as adjuvant medical therapy after surgery, although reported in 25% of the total treated cases, appears to imply a non-significant difference in the cumulative relapses considering the time to event analysis (see Supplemental Fig. 3 and Fig. 4C). In all cases, considered in these analysis (Supplemental Fig. 3 and Fig. 4C), the drug used was MPA. In 2 cases, 1 after first treatment and 1 after the appearance of the second recurrence, intrauterine device (IUD) medicated with levonergestrel was used, in both no recurrence was described.^[15,23]^ In addition, the group of Nomura published recently a second article describing the same cases of a previous case series considered in this meta-analysis, and showing that continuing in 5 cases with progestogens treatment after MPA treatment will reduce the incidence of recurrences.^[4,28]^ Considering what emerges from the literature, it can be concluded that medical therapy does not entail particular advantages, especially when using a hysteroscopic eradication treatment, unless it is associated with cancer or endometrial hyperplasia where the benefit of MPA treatment has already been described.

In particular, we want to point out some interesting considerations about follow-up: it was noted that recurrences occur even after several years, at 10 years the cumulative recurrences shows a 59.54% risk (CI.95: 31.88–75.61%) comparable to the studies with greater follow up periods such as Longacre et al^[19]^ or Chyoda et al^[13]^ (9–10 years). In addition we observed a significantly higher risk of persistence/relapse in case of DCP than in operative hysteroscopy (*P* < .05).

This certainly imposes the need to monitor these patients considering also the specific category of patients seeking for pregnancy. It is not possible, of course, to define a precise follow-up scheme based on strong evidence base medicine, in fact there are different disparities in this literature. However, it is possible to envisage closer checks initially and subsequently less intensive, using ultrasound examinations followed, if in suspicion of recurrence by hysteroscopy and biopsy or by directly performing hysteroscopy and biopsy according to the local organizational setting.

The association with endometrial cancer was 16% (CI.95 9–29%), concurrent with APA diagnosis in 12% and during follow-up in 14%. Respectively, the association with endometrial hyperplasia was 15% (total), 10% (concomitant with APA diagnosis), and 11% (during follow up). In our analysis the time-to-event cumulative occurrences were also high (endometrial cancer 59.91% at 14 years of follow-up—hyperplasia 27.27% at 14 years of follow-up). These prevalences are higher than those shown by Heatley.^[27]^ The prevalence found in our meta-analysis may also be superior in consideration of the type of treatment, where treatment by DCP often does not allow a good differential diagnosis in comparison to hysteroscopy (at the time of Heatley was more commons DCP than operative hysteroscopy). This could be also justified by the fact that in our analysis the prevalence uniformity of endometrial hyperplasia diagnosis was higher in hysteroscopic cases than DCP cases because the higher accuracy of hysteroscopic diagnosis.

The pathogenesis of endometrial carcinoma associated to APA is unknown: it is not clear if the endometrial cancer is developing in the context of APA or in the remaining adjacent or distant endometrium. Bakalianou et al^[29]^ describes one case of 36-year-old patient with presence of neoplasm foci within APA specimen, as well as Mittal et al,^[30]^ Sonoyama et al,^[31]^ and Nejković et al.^[32]^ Evaluating the time course, the risk of diagnosis of endometrial neoplasia is 59.91% (CI.95: 29.54–77.19%) at 14 years of follow-up. The risk of cancer development during follow-up according to the cumulative analysis is statistically reduced in cases treated primarily with hysteroscopic surgery (*P* < .05) than those treated with DCP. These data highlight the need for long-term follow-up. Furthermore, these same data invite us to reflect on what should be the treatment of the patient once the reproductive process has ended, or rather if there is any indication to remove the uterus after pregnancy or in case of recurrence. Despite this kind of management with histerectomy at the end of reproductive age does not change the long-time prognosis of these patients, it can have psychological repercussions.

Finally, the studies considering the pregnancy outcome are scarce, in fact pregnancy outcome is under-reported especially in less recent studies. Our meta-analysis shows among patients undergone to conservative surgical treatment a successful pregnancy in 78% of cases where there is a clear desire for offspring, however with a variability related to woman age or associated pathologies, or use of medically assisted reproduction (that could be a choice to shorten the time for having a pregnancy in a population at high risk of having recurrences or new endometrial cancer diagnosis).^[4]^

In summary APA is a rare lesion, which occurs predominantly in fertile age and in association with infertility and nulliparity. Conservative treatment is indicated in these patients. The data emerged from this review suggests that conservative treatment performed by operative histeroscopy (lesion resection and endometrial biopsies adjacent to the lesion and at a distance) is the optimal choice because it optimizes fertility preservation, it lowers the risk of recurrences and improve the accuracy of concomitant carcinoma or hyperplasia diagnosis. The medical treatment with MPA, following conservative surgery, does not appear to be beneficial in the treated cases, except in the presence of a diagnosis of carcinoma or endometrial hyperplasia.

## Acknowledgments

The authors would like to thank everyone who voluntary dedicated their time and effort.

## Author contributions

Substantial contributions to conception and design or acquisition of data or to analysis and interpretation of data (Anna Biasioli, Ambrogio P. Londero, Maria Orsaria, Federica Scrimin, Francesco Paolo Mangino, Serena Bertozzi, Laura Mariuzzi, Angelo Cagnacci). Drafting the article or revising it critically for important intellectual content (Anna Biasioli, Ambrogio P. Londero, Maria Orsaria, Federica Scrimin, Francesco Paolo Mangino, Serena Bertozzi, Laura Mariuzzi, Angelo Cagnacci). All authors have read and approved the final manuscript.

## Supplementary Material

Supplemental Digital Content

## Supplementary Material

Supplemental Digital Content

## Supplementary Material

Supplemental Digital Content

## Supplementary Material

Supplemental Digital Content

## Supplementary Material

Supplemental Digital Content

## Supplementary Material

Supplemental Digital Content

## Supplementary Material

Supplemental Digital Content

## Supplementary Material

Supplemental Digital Content

## Supplementary Material

Supplemental Digital Content

## Supplementary Material

Supplemental Digital Content

## Supplementary Material

Supplemental Digital Content

## Supplementary Material

Supplemental Digital Content

## Supplementary Material

Supplemental Digital Content
