# Supplementary material for: Atypical polypoid adenomyoma follow-up and management: Systematic review of case reports and series and meta-analysis
Source: Medicine (Baltimore). 2020 Jun 26;99(26):e20491. doi: 10.1097/MD.0000000000020491 (PMC7328951; doi:10.1097/MD.0000000000020491)

Supplemental Figure 1A- Summary diagram of the methodological quality analysis of the included studies (part 1). Color legend: green (+) item present (high quality), yellow (?) not clear, and red (-) item missing (low quality).

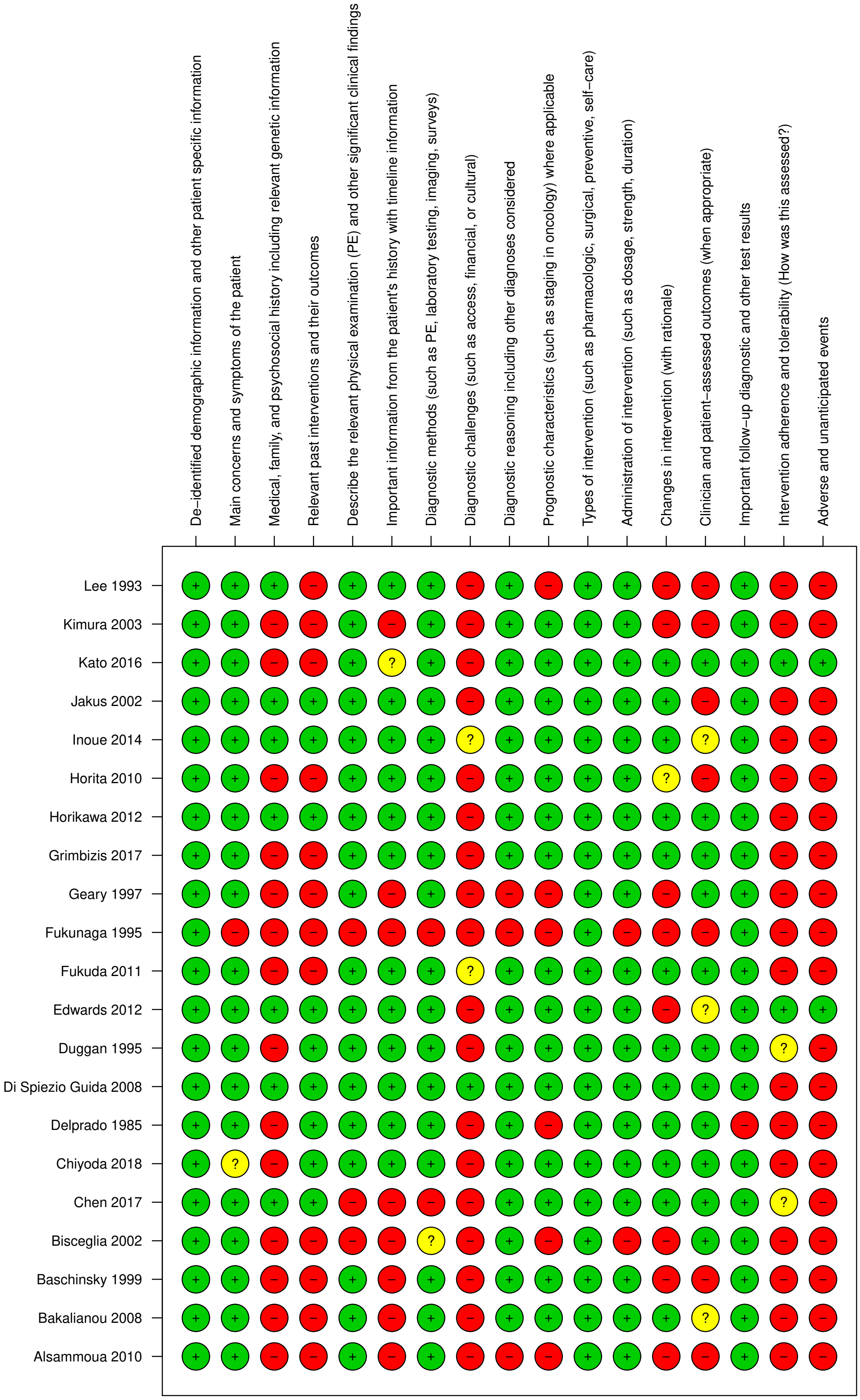

Supplement: Supplemental Digital Content [file medi-99-e20491-s001.pdf]
