# Supplementary material for: Atypical polypoid adenomyoma follow-up and management: Systematic review of case reports and series and meta-analysis
Source: Medicine (Baltimore). 2020 Jun 26;99(26):e20491. doi: 10.1097/MD.0000000000020491 (PMC7328951; doi:10.1097/MD.0000000000020491)
Supplement: Supplemental Digital Content [file medi-99-e20491-s002.pdf]

Supplemental Figure 1B- Summary diagram of the methodological quality analysis of the included studies (part 2). Color legend: green (+) item present (high quality), yellow (?) not clear, and red (-) item missing (low quality).

Study	De–identified demographic information and other patient specific information	Main concerns and symptoms of the patient	Medical, family, and psychosocial history including relevant genetic information	Relevant past interventions and their outcomes	Describe the relevant physical examination (PE) and other significant clinical findings	Important information from the patient's history with timeline information	Diagnostic methods (such as PE, laboratory testing, imaging, surveys)	Diagnostic challenges (such as access, financial, or cultural)	Diagnostic reasoning including other diagnoses considered	Prognostic characteristics (such as staging in oncology) where applicable	Types of intervention (such as pharmacologic, surgical, preventive, self–care)	Administration of intervention (such as dosage, strength, duration)	Changes in intervention (with rationale)	Clinician and patient–assessed outcomes (when appropriate)	Important follow–up diagnostic and other test results	Intervention adherence and tolerability (How was this assessed?)	Adverse and unanticipated events
Bo 2018	+	+	−	−	−	+	−	−	+	+	+	−	+	+	+	−	−
Narumi 2018	+	+	−	−	+	+	+	−	+	−	+	+	+	+	+	−	−
Nakabayashi 2018	−	+	+	+	+	−	+	+	+	+	+	+	−	−	−	−	−
Nemejcova 2015	+	+	−	−	−	−	+	−	?	+	+	+	−	+	+	−	−
Young 1986	+	+	−	−	+	−	+	−	−	−	+	+	−	+	+	−	−
Longacre 1996	+	+	−	+	+	+	+	+	+	+	+	+	+	+	+	+	+
Matsumoto 2013	+	+	+	+	+	+	+	−	+	+	+	+	+	+	+	−	−
Zhang 2012	+	+	−	+	+	+	+	−	+	?	+	+	+	+	+	−	−
Yamagami 2015	+	+	−	−	+	+	+	−	+	+	+	+	+	+	+	−	−
Yahata 2011	+	+	−	+	+	+	+	+	+	+	+	+	+	+	+	+	−
Wong 2007	+	+	+	−	+	+	+	−	+	+	+	+	−	+	+	−	−
Vilos 2003	+	+	−	−	+	−	+	−	+	+	+	+	+	−	+	−	+
Tziortziotis 1997	+	+	−	−	+	+	+	−	+	−	+	+	+	−	+	−	−
Tashiro 1998	+	+	−	−	+	−	+	−	+	+	+	+	+	+	+	−	−
Sonoyama 2014	+	+	−	−	+	?	+	−	+	+	+	+	−	−	+	−	−
Solima 2017	+	+	−	+	+	+	+	−	+	+	+	+	+	+	+	−	+
Rollason 1988	+	+	−	−	−	−	−	−	−	−	+	+	−	−	+	−	−
Ramos 2003	+	+	−	−	?	−	+	+	+	+	+	−	−	−	+	−	−
Protopapas 2016	+	+	−	−	+	?	?	?	?	+	+	+	+	?	+	−	−
Ohishi 2008	+	+	+	−	+	−	+	−	+	+	+	+	−	+	+	−	−
Nomura 2016	+	+	−	−	+	+	+	−	+	+	+	+	+	+	+	−	−
Nejkovic 2013	+	+	?	?	+	+	+	−	+	?	+	+	−	+	+	+	?
Mittal 1995	+	+	+	+	−	−	+	−	?	+	+	+	−	−	+	−	−
Mazur 1981	+	+	−	−	−	−	+	−	+	+	+	+	−	?	+	−	−
