# Supplementary material for: Atypical polypoid adenomyoma follow-up and management: Systematic review of case reports and series and meta-analysis
Source: Medicine (Baltimore). 2020 Jun 26;99(26):e20491. doi: 10.1097/MD.0000000000020491 (PMC7328951; doi:10.1097/MD.0000000000020491)

Supplemental Figure 2- Summary forest-plot of APA relapse prevalence without considering the specific time-to-event follow-up; in this case data were stratified by type of conservative treatment and by aggregated and not aggregated. Case series with less than five cases and individual case reports were aggregated.

Study	Rec.	Cases	Proportion	CI 95%	Weight
**Level = Non–aggregated – Histeroscopy**					
Chen 2017	1	10	0.10	[0.00; 0.45]	10.5%
Grimbizis 2017	2	9	0.22	[0.03; 0.60]	14.5%
**Random effects model**	**3**	**19**	**0.17**	**[0.05; 0.41]**	**25.0%**
Heterogeneity: $I^2 = 0\%$, $\chi^2_1 = 0.51$ ($p = 0.48$)					
**Level = Aggregated – Histeroscopy**					
Aggregated case reports 2003–2018	4	16	0.25	[0.07; 0.52]	19.3%
**Random effects model**	**4**	**16**	**0.25**	**[0.10; 0.51]**	**19.3%**
Heterogeneity: not applicable					
**Level = Non–aggregated – DCP**					
Fukunaga 1995	0	5	0.00	[0.00; 0.52]	6.5%
Nomura 2016	12	18	0.67	[0.41; 0.87]	21.2%
Ohishi 2008	1	5	0.20	[0.01; 0.72]	9.7%
**Random effects model**	**13**	**28**	**0.33**	**[0.07; 0.78]**	**37.3%**
Heterogeneity: $I^2 = 67\%$, $\chi^2_2 = 6.03$ ($p = 0.05$)					
**Level = Aggregated – DCP**					
Aggregated case reports 1981–2012	4	12	0.33	[0.10; 0.65]	18.4%
**Random effects model**	**4**	**12**	**0.33**	**[0.13; 0.62]**	**18.4%**
Heterogeneity: not applicable					
**Random effects model**	**24**	**75**	**0.29**	**[0.15; 0.48]**	**100.0%**
Heterogeneity: $I^2 = 52\%$, $\chi^2_6 = 12.38$ ($p = 0.05$)					

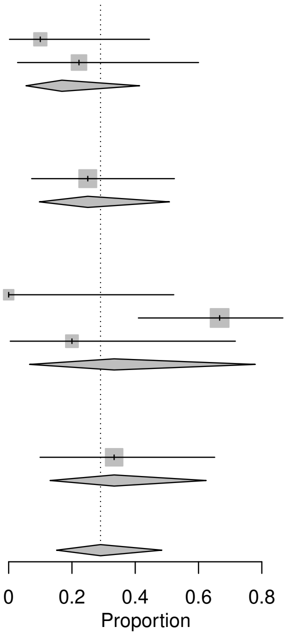

Supplement: Supplemental Digital Content [file medi-99-e20491-s003.pdf]
