# Supplementary material for: Atypical polypoid adenomyoma follow-up and management: Systematic review of case reports and series and meta-analysis
Source: Medicine (Baltimore). 2020 Jun 26;99(26):e20491. doi: 10.1097/MD.0000000000020491 (PMC7328951; doi:10.1097/MD.0000000000020491)

Supplemental Figure 3- Forest-plot of APA relapse prevalence without considering the specific time-to-event follow-up. Summary forest-plot we stratified between the cases where medical therapy after surgery was performed or not.

Study	Rec.	Cases	Proportion	CI 95%	Weight
**Level = No medical therapy**					
Fukunaga 1995	0	5	0.00	[0.00; 0.52]	7.9%
Grimbizis 2017	2	9	0.22	[0.03; 0.60]	17.0%
Aggregated case reports 1981–2018	7	23	0.30	[0.13; 0.53]	25.1%
**Random effects model**	**9**	**37**	**0.26**	**[0.14; 0.43]**	**50.0%**
Heterogeneity: $I^2 = 0\%$, $\chi^2_2 = 1.14$ ($p = 0.57$)					
**Level = Medical therapy**					
Chen 2017	1	10	0.10	[0.00; 0.45]	12.6%
Nomura 2016	12	18	0.67	[0.41; 0.87]	23.9%
Aggregated case reports 1981–2018	2	4	0.50	[0.07; 0.93]	13.4%
**Random effects model**	**15**	**32**	**0.42**	**[0.12; 0.79]**	**50.0%**
Heterogeneity: $I^2 = 68\%$, $\chi^2_2 = 6.16$ ($p = 0.05$)					
**Random effects model**	**24**	**69**	**0.33**	**[0.16; 0.55]**	**100.0%**
Heterogeneity: $I^2 = 57\%$, $\chi^2_5 = 11.59$ ($p = 0.04$)					

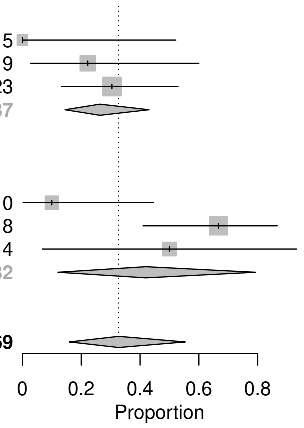

Supplement: Supplemental Digital Content [file medi-99-e20491-s004.pdf]
