# Supplementary material for: Atypical polypoid adenomyoma follow-up and management: Systematic review of case reports and series and meta-analysis
Source: Medicine (Baltimore). 2020 Jun 26;99(26):e20491. doi: 10.1097/MD.0000000000020491 (PMC7328951; doi:10.1097/MD.0000000000020491)

Supplemental Figure 4- Forest-plot of the prevalence of concomitant diagnosis of endometrial neoplasia divided by type of conservative treatment. The time-to-event follow-up data is not considered in this analysis. Case series with less than five cases and individual case reports were aggregated.

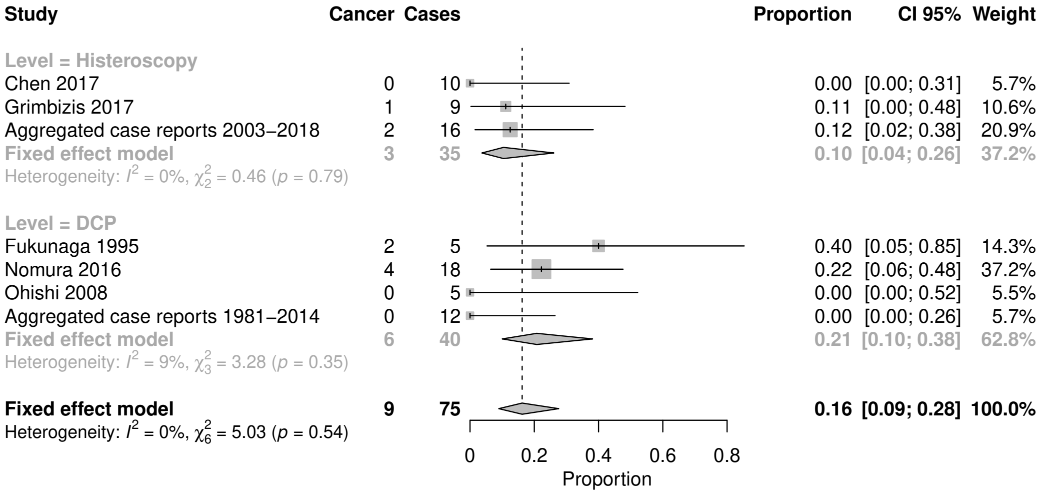

Supplement: Supplemental Digital Content [file medi-99-e20491-s005.pdf]
