# Supplementary material for: Atypical polypoid adenomyoma follow-up and management: Systematic review of case reports and series and meta-analysis
Source: Medicine (Baltimore). 2020 Jun 26;99(26):e20491. doi: 10.1097/MD.0000000000020491 (PMC7328951; doi:10.1097/MD.0000000000020491)

Supplemental Figure 5- Forest-plot of the prevalence of endometrial neoplasia diagnosis during follow-up (without considering time-to-event data). Cases with hysterectomy at diagnosis were excluded. Case series with less than five cases and individual case reports were aggregated.

Study	Cancer	Cases	Proportion	CI 95%	Weight
**Level = Aggregated**					
Aggregated case reports 1985–2018	6	24	0.25	[0.10; 0.47]	16.0%
**Random effects model**	**6**	**24**	**0.25**	**[0.12; 0.46]**	**16.0%**
Heterogeneity: not applicable					
**Level = Non–aggregated**					
Bo 2018	0	34	0.00	[0.00; 0.10]	5.7%
Chen 2017	1	10	0.10	[0.00; 0.45]	8.5%
Chiyoda 2018	7	35	0.20	[0.08; 0.37]	16.8%
Fukunaga 1995	0	5	0.00	[0.00; 0.52]	5.4%
Grimbizis 2017	1	9	0.11	[0.00; 0.48]	8.4%
Longacre 1996	0	44	0.00	[0.00; 0.08]	5.7%
Nemejcova 2015	0	6	0.00	[0.00; 0.46]	5.5%
Nomura 2016	9	18	0.50	[0.26; 0.74]	16.0%
Young 1986	2	23	0.09	[0.01; 0.28]	12.1%
**Random effects model**	**20**	**184**	**0.12**	**[0.05; 0.25]**	**84.0%**
Heterogeneity: $I^2 = 62\%$, $\chi^2_8 = 21.19$ ($p < 0.01$)					
**Random effects model**	**26**	**208**	**0.14**	**[0.07; 0.26]**	**100.0%**
Heterogeneity: $I^2 = 58\%$, $\chi^2_9 = 21.60$ ($p = 0.01$)					

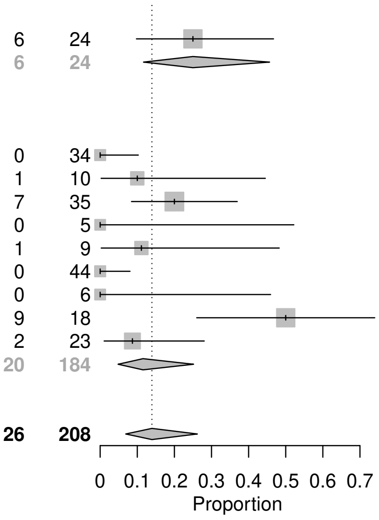

Supplement: Supplemental Digital Content [file medi-99-e20491-s006.pdf]
