# Supplementary material for: Atypical polypoid adenomyoma follow-up and management: Systematic review of case reports and series and meta-analysis
Source: Medicine (Baltimore). 2020 Jun 26;99(26):e20491. doi: 10.1097/MD.0000000000020491 (PMC7328951; doi:10.1097/MD.0000000000020491)

Supplemental Figure 7- Forest-plot of the prevalence of concomitant finding (at the time of APA diagnosis) of endometrial hyperplasia. The time-to-event follow-up is not considered in this analysis. Case series with less than five cases and individual case reports were aggregated.

Study	Hyper–plasia	Cases	Proportion	CI 95%	Weight
**Level = Aggregated**					
Aggregated case reports 1995–2018	4	36	0.11	[0.03; 0.26]	15.2%
**Random effects model**	**4**	**36**	**0.11**	**[0.04; 0.26]**	**15.2%**
Heterogeneity: not applicable					
**Level = Non–aggregated**					
Bo 2018	0	43	0.00	[0.00; 0.08]	6.5%
Chen 2017	5	10	0.50	[0.19; 0.81]	13.9%
Chiyoda 2018	2	35	0.06	[0.01; 0.19]	12.7%
Fukunaga 1995	1	6	0.17	[0.00; 0.64]	8.9%
Grimbizis 2017	2	9	0.22	[0.03; 0.60]	11.9%
Longacre 1996	0	55	0.00	[0.00; 0.06]	6.5%
Mazur 1981	1	5	0.20	[0.01; 0.72]	8.7%
Nemejcova 2015	0	13	0.00	[0.00; 0.25]	6.3%
Nomura 2016	1	18	0.06	[0.00; 0.27]	9.5%
**Random effects model**	**12**	**194**	**0.10**	**[0.04; 0.24]**	**84.8%**
Heterogeneity: $I^2 = 61\%$, $\chi^2_8 = 20.4$ ($p < 0.01$)					
**Random effects model**	**16**	**230**	**0.10**	**[0.05; 0.22]**	**100.0%**
Heterogeneity: $I^2 = 56\%$, $\chi^2_9 = 20.54$ ($p = 0.01$)					

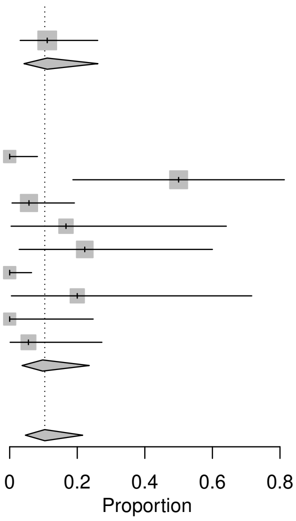

Supplement: Supplemental Digital Content [file medi-99-e20491-s008.pdf]
