# Supplementary material for: Atypical polypoid adenomyoma follow-up and management: Systematic review of case reports and series and meta-analysis
Source: Medicine (Baltimore). 2020 Jun 26;99(26):e20491. doi: 10.1097/MD.0000000000020491 (PMC7328951; doi:10.1097/MD.0000000000020491)

Supplemental Figure 8- Forest-plot of endometrial hyperplasia diagnosis prevalence during the follow-up (without considering time-to-event data). Cases with hysterectomy at diagnosis were excluded. Case series with less than five cases and individual case reports were aggregated.

Study	Hyper-plasia	Cases	Proportion	CI 95%	Weight
**Level = Aggregated**					
Aggregated case reports 1995–2018	1	24	0.04	[0.00; 0.21]	8.3%
**Fixed effect model**	1	24	0.04	[0.01; 0.24]	8.3%
Heterogeneity: not applicable					
**Level = Non–aggregated**					
Bo 2018	2	34	0.06	[0.01; 0.20]	16.3%
Chen 2017	4	10	0.40	[0.12; 0.74]	20.7%
Chiyoda 2018	4	35	0.11	[0.03; 0.27]	30.6%
Fukunaga 1995	0	5	0.00	[0.00; 0.52]	4.0%
Grimbizis 2017	1	9	0.11	[0.00; 0.48]	7.7%
Longacre 1996	0	44	0.00	[0.00; 0.08]	4.3%
Nemejcova 2015	0	6	0.00	[0.00; 0.46]	4.0%
Nomura 2016	0	18	0.00	[0.00; 0.19]	4.2%
**Fixed effect model**	11	161	0.12	[0.07; 0.19]	91.7%
Heterogeneity: $I^2 = 41\%$, $\chi^2_7 = 11.78$ ($p = 0.11$)					
**Fixed effect model**	**12**	**185**	**0.11**	**[0.06; 0.18]**	**100.0%**
Heterogeneity: $I^2 = 38\%$, $\chi^2_8 = 12.85$ ($p = 0.12$)					

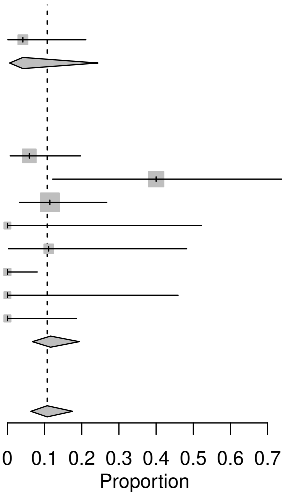

Supplement: Supplemental Digital Content [file medi-99-e20491-s009.pdf]
