# Supplementary material for: Atypical polypoid adenomyoma follow-up and management: Systematic review of case reports and series and meta-analysis
Source: Medicine (Baltimore). 2020 Jun 26;99(26):e20491. doi: 10.1097/MD.0000000000020491 (PMC7328951; doi:10.1097/MD.0000000000020491)
Supplement: Supplemental Digital Content [file medi-99-e20491-s011.pdf]

Supplemental List 1- List of included and excluded articles after full-text review.

Included studies

Alsammoua 2010
Alsammoua, S.; Teoh, P. J. & Khalifa, L. (2010), 'Atypical polypoid adenomyoma.', *Journal of obstetrics and gynaecology : the journal of the Institute of Obstetrics and Gynaecology* **30**, 419--420.

Bakalianou 2008
Bakalianou, K.; Salakos, N.; Iavazzo, C.; Paltoglou, G.; Papadias, K. & Kondi-Pafiti, A. (2008), 'A case of endometrial carcinoma arising in a 36-year-old woman with uterine atypical polypoid adenomyoma (APA).', *Eur J Gynaecol Oncol* **29**, 298--299.

Baschinsky 1999
Baschinsky, D.; Keyhani-Rofagha, S. & Hameed, A. (1999), 'Exfoliative cytology of atypical polypoid adenomyoma. A case report.', *Acta Cytol* **43**, 637--640.

Bisceglia 2002
Bisceglia, M. (2002), 'Atypical polypoid adenomyoma.', *Adv Anat Pathol* **9**, 256–260.

Bo 2018
Ma, B. Zhu, Y. & Liu, Y. (2018), ‘Management of atypical polypoid adenomyoma of the uterus: A single center's experience.’, *Medicine (Baltimore)* **97**, e0135.

Chen 2017
Chen, Q.; Lu, W. & Lu, B. (2017), 'Pregnant outcomes of atypical polypoid adenomyoma treated with progestin therapy.', *The journal of obstetrics and gynaecology research*.

Chiyoda 2018
Chiyoda, T.; Lin, B.-L.; Saotome, K.; Kiyokawa, S. & Nakada, S. (2018), 'Hysteroscopic Transcervical Resection for Atypical Polypoid Adenomyoma of the Uterus: A Valid, Fertility-Preserving Option.', *J Minim Invasive Gynecol* **25**, 163--169.e1.

Delprado 1985
Delprado, W. J.; Stevens, S. M. & Baird, P. J. (1985), 'Atypical polypoid adenomyoma: a case report with ultrastructural examination.', *Pathology (Phila)* **17**, 522--525.

Di Spiezio/Guida 2008
Di Spiezio Sardo, A.; Mazzon, I.; Gargano, V.; Di Carlo, C.; Guida, M.; Mignogna, C.; Bifulco, G. & Nappi, C. (2008), 'Hysteroscopic treatment of atypical polypoid adenomyoma diagnosed incidentally in a young infertile woman.', *Fertil Steril* **89**, 456.e9--456.12.

Guida, M.; Maurizio, G.; Greco, E.; Di Spiezio Sardo, A.; Di Carlo, C.; Lavitola, G.; Tarsitano, F. & Nappi, C. (2008), 'Successful pregnancy after four-step hysteroscopic technique for the treatment of atypical polypoid adenomyoma.', *Fertil Steril* **89**, 1283--1284.

Duggan 1995
Duggan, M. A.; Rowlands, C.; Kneafsey, P. D.; Nation, J. G. & Stuart, G. C. (1995), 'Uterine atypical polypoid adenomyoma and ovarian endometrioid carcinoma: metastatic disease or dual primaries?', *International journal of gynecological pathology : official journal of the International Society of Gynecological Pathologists* **14**, 81--86.

Edwards 2012
Edwards, J. M.; Alsop, S. & Modesitt, S. C. (2012), 'Coexisting atypical polypoid adenomyoma and endometrioid endometrial carcinoma in a young woman with Cowden Syndrome: Case report and implications for screening and prevention.', *Gynecologic oncology case reports* **2**, 29--31.

Fukuda 2011
Fukuda, M.; Sakurai, N.; Yamamoto, Y.; Taoka, H.; Asakawa, Y.; Kurasaki, A.; Oharaseki, T.; Takahashi, K. & Kubushiro, K. (2011), 'Case of atypical polypoid adenomyoma that possibly underwent a serial progression from endometrial hyperplasia to carcinoma.', *The journal of obstetrics and gynaecology research* **37**, 468--471.

Fukunaga 1995
Fukunaga, M.; Endo, Y.; Ushigome, S. & Ishikawa, E. (1995), 'Atypical polypoid adenomyomas of the uterus.', *Histopathology* **27**, 35--42.

Geary 1997
Geary, M.; Lenehan, P. & Kelehan, P. (1997), 'Recurrent atypical polypoid adenomyoma in association with ovarian carcinoma-a cautionary tale.', *Journal of obstetrics and gynaecology : the journal of the Institute of Obstetrics and Gynaecology* **17**, 502.

Grimbizis 2017
Grimbizis, G. F.; Mikos, T.; Miliaras, D.; Kioussis, G.; Theodoridis, T. D.; Tsolakidis, D. & Tarlatzis, B. C. (2017), 'Management of atypical polypoid adenomyomas. A case series.', *Eur J Obstet Gynecol Reprod Biol* **215**, 1--5.

Horikawa 2012
Horikawa, M.; Shinmoto, H.; Soga, S.; Shiomi, E.; Sei, K.; Shimazaki, H. & Kaji, T. (2012), 'Multiple atypical polypoid adenomyoma of the uterus.', *Japanese journal of radiology* **30**, 606--611.

Horita 2010
Horita, A.; Kurata, A.; Komatsu, K.; Yajima, M. & Sakamoto, A. (2010), 'Coexistent atypical polypoid adenomyoma and complex atypical endometrial hyperplasia in the uterus.', *Diagn Cytopathol* **38**, 527--532.

Inoue 2014
Inoue, K.; Tsubamoto, H.; Hori, M.; Ogasawara, T. & Takemura, T. (2014), 'A case of endometrioid adenocarcinoma developing 8 years after conservative management for atypical polypoid adenomyoma.', *Gynecologic oncology case reports* **8**, 21--23.

Jakus 2002
Jakus, S.; Edmonds, P.; Dunton, C. & Holland, G. (2002), 'Atypical polypoid adenomyoma mimicking cervical adenocarcinoma.', *Journal of lower genital tract disease* **6**, 33--38.

Kato 2016
Kato, Y.; Kiyomizu, M.; Watanabe, A.; Takehara, Y.; Ogawa, K. & Suganuma, I. (2016), 'A mixed carcinoma of the uterus arising from an atypical polypoid adenomyoma: A case report', *Eur J Gynaecol Oncol* **37**(6), 864-866.

Kimura 2003
Kimura, J.; Okamoto, H.; Yamamoto, H.; Mazaki, T. & Kitamura, T. (2003), 'Cytologic features of

atypical polypoid adenomyoma of the endometrium. A case report.', *Acta Cytol* **47**, 287--292.

Lee 1993
Lee, K. R. (1993), 'Atypical polypoid adenomyoma of the endometrium associated with adenomyomatosis and adenocarcinoma.', *Gynecol Oncol* **51**, 416--418.

Longacre 1996
Longacre, T. A.; Chung, M. H.; Rouse, R. V. & Hendrickson, M. R. (1996), 'Atypical polypoid adenomyofibromas (atypical polypoid adenomyomas) of the uterus. A clinicopathologic study of 55 cases.', *The American journal of surgical pathology* **20**, 1--20.

Matsumoto 2013
Matsumoto, T.; Hiura, M.; Baba, T.; Ishiko, O.; Shiozawa, T.; Yaegashi, N.; Kobayashi, H.; Yoshikawa, H.; Kawamura, N. & Kaku, T. (2013), 'Clinical management of atypical polypoid adenomyoma of the uterus. A clinicopathological review of 29 cases.', *Gynecol Oncol* **129**, 54--57.

Mazur 1981
Mazur, M. T. (1981), 'Atypical polypoid adenomyomas of the endometrium.', *The American journal of surgical pathology* **5**, 473--482.

Mittal 1995
Mittal, K. R.; Peng, X. C.; Wallach, R. C. & Demopoulos, R. I. (1995), 'Coexistent atypical polypoid adenomyoma and endometrial adenocarcinoma.', *Hum Pathol* **26**, 574--576.

Nakabayashi 2018
Nakabayashi, A.; Takahashi, N.; Hashimoto, K.; Nagata, R.; Hashimoto, T.; Yamamoto, T.; & Matsui H. (2018), 'A case of rapidly-growing atypical polypoid adenomyoma which was histologically diagnosed before operation and removed by a laparoscopic resection.', *Taiwan J Obstet Gynecol* **57**,115-118.

Narumi 2018
Narumi, R.; Takei, Y.; Morisawa, H.; Taneichi, A.; Matsubara, S.; & Fujiwara, H. (2019), 'Successful laparotomy tumor resection and levonorgestrel-releasing intrauterine system for atypical polypoid adenomyoma.', J Obstet Gynaecol Res **45**, 230-234.

Nejkovic 2013
Nejković, L.; Pazin, V. & Filimonović, D. (2013), 'Atypical polypoid adenomyoma mixed with endometrioid carcinoma: a case report.', *Eur J Gynaecol Oncol* **34**, 101--103.

Nemejcova 2015
Němejcová, K.; Kenny, S. L.; Laco, J.; Škapa, P.; Staněk, L.; Zikán, M.; Kleiblová, P.; McCluggage, W. G. & Dundr, P. (2015), 'Atypical Polypoid Adenomyoma of the Uterus: An Immunohistochemical and Molecular Study of 21 Cases.', *The American journal of surgical pathology* **39**, 1148--1155.

Nomura 2016
Nomura, H.; Sugiyama, Y.; Tanigawa, T.; Matoda, M.; Kanao, H.; Kondo, E. & Takeshima, N. (2016), 'Long-term outcomes of fertility-sparing treatment of atypical polypoid adenomyoma with medroxyprogesterone acetate.', *Arch Gynecol Obstet* **293**, 177--181.

Ohishi 2008
Ohishi, Y.; Kaku, T.; Kobayashi, H.; Aishima, S.; Umekita, Y.; Wake, N. & Tsuneyoshi, M. (2008),

'CD10 immunostaining distinguishes atypical polypoid adenomyofibroma (atypical polypoid adenomyoma) from endometrial carcinoma invading the myometrium.', *Hum Pathol* **39**, 1446--1453.

Protopapas 2016
Protopapas, A.; Sotiropoulou, M.; Athanasiou, S. & Loutradis, D. (2016), 'Endocervical Atypical Polypoid Adenomyoma.', *J Minim Invasive Gynecol* **23**, 130--132.

Ramos 2003
Ramos, P.; Valenzuela, P.; Santana, A.; Ruiz, A. & Solano, J. (2003), 'Atypical polypoid adenomyoma of the uterine cervix: a diagnostic problem.', *Journal of obstetrics and gynaecology : the journal of the Institute of Obstetrics and Gynaecology* **23**, 319--321.

Rollason 1988
Rollason, T. P. & Redman, C. W. (1988), 'Atypical polypoid adenomyoma–clinical histological and immunocytochemical findings.', *Eur J Gynaecol Oncol* **9**, 444--451.

Solima 2017
Solima, E.; Liprandi, V.; Belloni, G. M.; Vignali, M. & Busacca, M. (2017), 'Recurrent atypical polypoid adenomyoma and pregnancy: A new conservative approach with levonorgestrel-releasing intrauterine system.', *Gynecologic oncology reports* **21**, 84--85.

Sonoyama 2014
Sonoyama, A.; Kanda, M.; Ojima, Y.; Kizaki, T. & Ohara, N. (2014), 'Coexistence of endometrioid adenocarcinoma in atypical polypoid adenomyoma.', *The Kobe journal of medical sciences* **60**, E74--E77.

Tashiro 1998
Tashiro, H.; Katabuchi, H.; Fukumatsu, Y.; Tanaka, N.; Yamashita, Y.; Matsuura, K. & Okamura, H. (1998), 'Endometrial adenocarcinoma following the conservative treatment of an atypical polypoid adenomyoma', *Int J Clin Oncol* **3**(6), 400-402.

Tziortziotis 1997
Tziortziotis, D. & Mortakis, A. (1997), 'Atypical polypoid adenomyoma of the uterus.', *Journal of obstetrics and gynaecology : the journal of the Institute of Obstetrics and Gynaecology* **17**, 110--111.

Vilos 2003
Vilos, G. A. & Ettler, H. C. (2003), 'Atypical polypoid adenomyoma and hysteroscopic endometrial ablation.', *Journal of obstetrics and gynaecology Canada : JOGC = Journal d'obstetrique et gynecologie du Canada : JOGC* **25**, 760--762.

Wong 2007
Wong, A. Y. K.; Chan, K.-s.; Lau, W.-l. & Tang, L. C. H. (2007), 'Pregnancy outcome of a patient with atypical polypoid adenomyoma.', *Fertil Steril* **88**, 1438.e7--1438.e9.

Yahata 2011
Yahata, T.; Nonaka, T.; Watanabe, A.; Sekine, M. & Tanaka, K. (2011), 'Complete hysteroscopic resection of a large atypical polypoid adenomyoma, followed by a successful pregnancy.', *Fertil Steril* **95**, 2435.e9--2435.11.

Yamagami 2015

Yamagami, W.; Susumu, N.; Ninomiya, T.; Nakadaira, N.; Iwasa, N.; Kuwahata, M.; Nomura, H.; Kataoka, F.; Banno, K. & Aoki, D. (2015), 'Hysteroscopic transcervical resection is useful to diagnose myometrial invasion in atypical polypoid adenomyoma coexisting with atypical endometrial hyperplasia or endometrial cancer with suspicious myometrial invasion.', *The journal of obstetrics and gynaecology research* **41**, 768--775.

Young 1986
Young, R. H.; Treger, T. & Scully, R. E. (1986), 'Atypical polypoid adenomyoma of the uterus. A report of 27 cases.', *Am J Clin Pathol* **86**, 139--145.

Zhang 2012
Zhang, H. K. & Chen, W. D. (2012), 'Atypical polypoid adenomyomas progressed to endometrial endometrioid adenocarcinomas.', *Arch Gynecol Obstet* **286**, 707--710.

Excluded articles

Reasons for exclusion: non-English language; selected outcomes were not present (e.g. histerctomy with presence or absence of concomitant pre-malignant or malignant lesions), non reported the clinical data or follow up (e.g. articles only about imaging or pathologic diagnosis); review; post-menopausal cases.

1. Buénerd, A.; Dargent, D.; Scoazec, J.-Y. & Berger, G. (2003), '[Carcinomatous transformation of an atypical polypoid adenomyofibroma of the uterus].', *Ann Pathol* **23**, 63--66.
2. Cheng, C.-S.; Wei, Y.-C.; Chu, T.-Y. & Ding, D.-C. (2016), 'Serous adenocarcinoma of endometrium coexistence with atypical polypoid adenomyoma in a postmenopausal woman: A rare case', *Journal of Medical Sciences (Taiwan)* **36**(4), 162-164.
3. Clouqueur, E.; Lucot, J. P.; Collinet, P.; Farine, M. O.; Kerdraon, O. & Poncelet, E. (2014), '[Atypical polypoid adenomyoma: retrospective study about 8 cases from Jeanne-de-Flandre Hospital between 1996 and 2008].', *Gynecologie, obstetrique & fertilite* **42**, 84--91.
4. D'Angelo, E. & Prat, J. (2011), 'Pathology of mixed Müllerian tumours.', *Best practice & research. Clinical obstetrics & gynaecology* **25**, 705--718.
5. Dinas, K.; Daniilidis, A.; Drizis, E.; Zaraboukas, T. & Tzafettas, J. (2009), 'Incidental diagnosis of atypical polypoid adenomyoma in a young infertile woman.', *Eur J Gynaecol Oncol* **30**, 701--703.
6. Fukami, T.; Yoshikai, T.; Tsujioka, H.; Tohyama, A.; Sorano, S.; Matsuoka, S.; Yamamoto, H.; Nakamura, S.; Goto, M.; Matsuoka, R.; Oya, M.; Torii, Y. & Eguchi, F. (2016), 'Positron Emission Tomography Findings in Atypical Polypoid Adenomyoma.', *Rare tumors* **8**, 6129.
7. Grevenkamp, F.; Kommoss, F.; Kommoss, F.; Lax, S.; Fend, F.; Wallwiener, D.; Schönfisch, B.; Krämer, B.; Brucker, S. Y.; Taran, F.-A.; Staebler, A. & Kommoss, S. (2017), 'Second Opinion Expert Pathology in Endometrial Cancer: Potential Clinical Implications.', *International journal of gynecological cancer : official journal of the International Gynecological Cancer Society* **27**, 289--296.
8. Heatley, M. K. (2006), 'Atypical polypoid adenomyoma: a systematic review of the English literature.', *Histopathology* **48**, 609--610.
9. Horita, A.; Kurata, A.; Maeda, D.; Fukayama, M. & Sakamoto, A. (2011), 'Immunohistochemical characteristics of atypical polypoid adenomyoma with special reference to h-caldesmon.', *International journal of gynecological pathology : official journal of the International Society of Gynecological Pathologists* **30**, 64--70.
10. Horn, L.-C.; Fischer, U. & Höckel, M. (2002), 'Polypoid endometrial adenomyoma in a postmenopausal woman, mimicking cervical cancer, associated with ovarian thecoma.', *Arch Gynecol Obstet* **267**, 101--103.
11. Jiang, Q.-Y.; Wang, L. & Wu, R.-J. (2013), 'A multiple perspectives on atypical polypoid adenomyoma of uterus.', *Gynecological endocrinology : the official journal of the International Society of Gynecological Endocrinology* **29**, 623--625.
12. Kenny, S. L. & McCluggage, W. G. (2014), 'Adenomyomatous Polyp of the Endometrium With Prominent Epithelioid Smooth Muscle Differentiation: Report of Two Cases of a Hitherto Undescribed Lesion.', *International journal of surgical pathology* **22**, 358--363.
13. Konstantinos, K.; Georgios, D.; Anysia, S. & Alexandros, R. (2017), 'The role of three-dimensional power Doppler hysterosonography (3-DPDS) in distinguishing atypical polypoid adenomyomas (APAs) from other intrauterine tumors: correlation with pathologic findings.', *Arch Gynecol Obstet* **296**, 391--396.
14. Kozawa, E.; Takahashi, M.; Meguro, S.; Yasuda, M.; Iwasa, N.; Fujiwara, K. & Kimura, F. (2013), 'Benign and malignant tumor of the uterine body with broccoli sign: MR imaging features for differential diagnosis.', *Japanese journal of radiology* **31**, 437--443.
15. Lee, E. J.; Han, J. H. & Ryu, H. S. (2004), 'Polypoid adenomyomas: sonohysterographic and color Doppler findings with histopathologic correlation.', *Journal of ultrasound in*

medicine : official journal of the American Institute of Ultrasound in Medicine **23**, 1421--9; quiz 1431.
16. Maeda, T.; Tateishi, U.; Sasajima, Y.; Hasegawa, T.; Daisaki, H.; Arai, Y. & Sugimura, K. (2006), 'Atypical polypoid adenomyoma of the uterus: appearance on (18)F-FDG PET/MRI fused images.', *AJR. American journal of roentgenology* **186**, 320--323.
17. McCluggage, W. G. (2006), 'My approach to the interpretation of endometrial biopsies and curettings.', *J Clin Pathol* **59**, 801--812.
18. Nakai, G.; Kitano, R.; Yamamoto, K.; Higashiyama, A.; Juri, H.; Tsuboyama, T.; Yamamoto, K.; Yamada, T.; Hirose, Y.; Ohmichi, M. & Narumi, Y. (2015), 'Magnetic resonance imaging findings in atypical polypoid adenomyoma.', *J Comput Assisted Tomogr* **39**, 32--36.
19. Nasu, K.; Miyazaki, T.; Takai, N. & Miyakawa, I. (2001), 'Atypical polypoid adenomyoma in a patient with hyperprolactinemia.', *International journal of gynecological cancer : official journal of the International Gynecological Cancer Society* **11**, 326--328.
20. Ota, S.; Catasus, L.; Matias-Guiu, X.; Bussaglia, E.; Lagarda, H.; Pons, C.; Muñoz, J.; Kamura, T. & Prat, J. (2003), 'Molecular pathology of atypical polypoid adenomyoma of the uterus.', *Hum Pathol* **34**, 784--788.
21. Sugiyama, T.; Nishida, T.; Hasuo, Y.; Kataoka, A. & Yakushiji, M. (1997), 'Atypical polypoid adenomyoma of the uterus: clinical and histological findings of three postmenopausal cases.', *Journal of obstetrics and gynaecology : the journal of the Institute of Obstetrics and Gynaecology* **17**, 304--306.
22. Sugiyama, T.; Ohta, S.; Nishida, T.; Okura, N.; Tanabe, K. & Yakushiji, M. (1998), 'Two cases of endometrial adenocarcinoma arising from atypical polypoid adenomyoma.', *Gynecol Oncol* **71**, 141--144.
23. Tahlan, A.; Nanda, A. & Mohan, H. (2006), 'Uterine adenomyoma: a clinicopathologic review of 26 cases and a review of the literature.', *International journal of gynecological pathology : official journal of the International Society of Gynecological Pathologists* **25**, 361--365.
24. Takahashi, H.; Yoshida, T.; Matsumoto, T.; Kameda, Y.; Takano, Y.; Tazo, Y.; Inoue, H. & Saegusa, M. (2014), 'Frequent β-catenin gene mutations in atypical polypoid adenomyoma of the uterus.', *Hum Pathol* **45**, 33--40.
25. Takeuchi, M.; Matsuzaki, K. & Harada, M. (2015), 'MR manifestations of uterine polypoid adenomyoma.', *Abdom Imaging* **40**, 480--487.
26. Terada, T. (2011), 'Atypical polypoid adenomyoma of the uterus: an immunohistochemical study on 5 cases.', *Ann Diagn Pathol* **15**, 338--341.
27. Yuan, C.-T.; Huang, W.-C.; Lee, C.-H.; Lin, M.-C.; Lee, C.-H.; Kao, Y.-C.; Huang, H.-Y.; Kuo, K.-T. & Lee, J.-C. (2017), 'Comprehensive screening for MED12 mutations in gynaecological mesenchymal tumours identified morphologically distinctive mixed epithelial and stromal tumours.', *Histopathology* **70**, 954--965.
28. Zhang, Z.-M.; Li, X.; Yang, X.-M.; Zhang, M.-J.; Xiao, S.; Kang, S. & Shan, B.-E. (2013), 'Analysis of factors associated with endometrial diseases in postoperative breast cancer patients receiving tamoxifen therapy', *Tumor* **33**(7), 629-633.
29. Zizi-Sermpetzoglou, A.; Moustou, E.; Petrakopoulou, N.; Arkoumani, E.; Tepelenis, N. & Savvaidou, V. (2012), 'Atypical polypoid adenomyoma of the uterus. A case report and a review of the literature.', *Eur J Gynaecol Oncol* **33**, 118–121.
30. Strickland, KC.; Yuan, L.; Quade, BJ.; Nucci, MR. & Howitt, BE. (2018), 'Clinicopathological and immunohistochemical features of uterine adenomyomatous polyps.' *Hum Pathol* **84**, 239-245.
31. McCluggage, WG. & Van de Vijver, K. (2018), 'ASATB2 is Consistently Expressed in Squamous Morules Associated With Endometrioid Proliferative Lesions and in the Stroma of Atypical Polypoid Adenomyoma.', *Int J Gynecol Pathol* doi: 10.1097/PGP.0000000000000544.
32. Nomura, H.; Sugiyama, Y.; Tanigawa, T.; Matoda, M.; Okamoto, S.; Omatsu, K.; Kanao, H.;

Kato, K.; Utsugi, K. & Takeshima, N. (2018), 'Maintenance hormonal therapy after treatment with medroxyprogesterone acetate for patients with atypical polypoid adenomyoma.', *Jpn J Clin Oncol* **48**, 255-258.
33. Chen, Q.; Lu, W. & Lu B. (2018), 'Pregnant outcomes of atypical polypoid adenomyoma treated with progestin therapy.', *J Obstet Gynaecol Res* **44,** 323-330.
