# Supplementary material for: Atypical polypoid adenomyoma follow-up and management: Systematic review of case reports and series and meta-analysis
Source: Medicine (Baltimore). 2020 Jun 26;99(26):e20491. doi: 10.1097/MD.0000000000020491 (PMC7328951; doi:10.1097/MD.0000000000020491)
Supplement: Supplemental Digital Content [file medi-99-e20491-s012.pdf]

Supplemental Table 1- Questions underlying this review.

1	In APA affected woman of childbearing age, what is the effect of conservative treatment on disease recurrence, pre-neoplastic lesions, cancer (concomitant or diagnosed during follow up), and pregnancy?
2	In APA affected woman of childbearing age, what is the effect of conservative operative hysteroscopic treatment compared to other conservative treatments or hysterectomies on disease recurrence, pre-neoplastic lesions, and cancer?
