# Supplementary material for: Atypical polypoid adenomyoma follow-up and management: Systematic review of case reports and series and meta-analysis
Source: Medicine (Baltimore). 2020 Jun 26;99(26):e20491. doi: 10.1097/MD.0000000000020491 (PMC7328951; doi:10.1097/MD.0000000000020491)
Supplement: Supplemental Digital Content [file medi-99-e20491-s013.pdf]

Supplemental Table 2- Characteristics of the included studies. (*) From the available data they appear to be two different case reports.

Study	Location	Cases number	Single case information extraction
Alsammoua 2010	Dumfries and Galloway Royal Infirmary - UK	1	Yes
Bakalianou 2008	University of Athens – Greece (*)	1	Yes
Baschinsky 1999	Ohio State University - USA	1	Yes
Bisceglia 2002	San Giovanni Rotondo - Italy	1	Yes
Bo 2018	Central Hospital of Obstetrics and GynecologyTianjin-China	43	No
Chen 2017	Zhejiang University - China	10	Yes
Chiyoda 2018	Kawasaki Municipal Hospital - Japan	35	Partially
Delprado 1985	Institute of Medical Pathology And Clinical Reasearch, Weastmead Hospital, Weastmead, Australia	1	Yes
Di Spiezio/Guida 2008	University of Naples Federico II - Italy	1	Yes
Duggan 1995	Foothills Hospital Calgary - Canada	1	Yes
Edwards 2012	Durham and Portsmouth and Charlottesville - USA	1	Yes
Fukuda 2011	Toho University School of Medicine - Japan	1	Yes
Fukunaga 1995	Jikei University School of Medicine - Japan	6	Yes
Geary 1997	National Maternity Hospital Dublin - Ireland	1	Yes
Grimbizis 2017	Aristotle University of Thessaloniki - Greece	9	Yes
Horikawa 2012	National Defense Medical College - Japan	1	Yes
Horita 2010	Kyorin University School of Medicine - Japan	1	Yes
Inoue 2014	Meiwa General Hospital/Hyogo College of Medicine/Okubo Hospital/Takemura Ladies Clinic – Japan	1	Yes
Jakus 2002	Thomas Jefferson University - USA	1	Yes
Kato 2016	Saiseikai Kyoto Hospital - Japan	1	Yes
Kimura 2003	National Maizuru Hospital - Japan	1	Yes
Lee 1993	University of Vermont - USA	1	Yes
Longacre 1996	Stanford University Medical Center - USA	55	No
Matsumoto 2013	Shikoku Cancer Center - Japan	29	No
Mazur 1981	Northwestern Univeristy - USA	5	Yes
Mittal 1995	New York University Medical Center - USA	1	Yes
Nakabayashi 2018	Tokyo Women's Medical University, Tokyo, Japan	1	Yes
Narumi 2018	Jichi Medical University-Tochigi- Japan	1	Yes
Nejkovic 2013	University of Belgrade - Serbia	1	Yes
Nemejcova 2015	Charles University in Prague - Czech Republic	13	No
Nomura 2016	Cancer Institute Hospital Ariake - Japan	18	Yes
Ohishi 2008	Kyushu University - Japan	7	Yes
Protopapas 2016	University of Athens – Greece (*)	1	Yes
Ramos 2003	Principe de Asturias University Hospital – Spain	1	Yes
Rollason 1988	University of Birmingham - UK	4	Yes
Solima 2017	University of Milan - Italy	1	Yes
Sonoyama 2014	Sanda Municipal Hospital – Japan	1	Yes
Tashiro 1998	Kumamoto Univ. School of Medicine – Japan	1	Yes
Tziortziotis 1997	LITO Hospital - Greece	1	Yes
Vilos 2003	The University of Western Ontario - Canada	1	Yes
Wong 2007	Kwong Wah Hospital - China	1	Yes
Yahata 2011	Niigata University Medical and Dental Hospital - Japan	1	Yes
Yamagami 2015	Keio University School of Medicine - Japan	3	Yes

Study	Location	Cases number	Single case information extraction
Young 1986	Massachusetts General Hospital - USA	27	No
Zhang 2012	Beijing Fuxing Hospital – China	2	Yes
